# Reduction of truncated Kit Expression in Men with Abnormal Semen Parameters, Globozoospermia and History of Low or Fertilization Failure

**DOI:** 10.22074/cellj.2019.6112

**Published:** 2019-06-15

**Authors:** Somayeh Haghighat, Marziyeh Tavalaee, Zahra Zakeri, Mahdi Noureddini, Abdolhossein Shahverdi, Mohammad Hossein Nasr Esfahani

**Affiliations:** 1 Physiology Research Center, Kashan University of Medical Sciences, Kashan, Iran; 2 Department of Reproductive Biotechnology, Reproductive Biomedicine Research Center, Royan Institute for Biotechnology, ACECR, Isfahan, Iran; 3 Department of Biology, Queens College and Graduate Center of the City University of New York, Flushing, NY, USA; 4 Department of Embryology, Reproductive Biomedicine Research Center, Royan Institute for Reproductive Medicine, ACECR, Tehran, Iran; 5 Isfahan Fertility and Infertility Center, Isfahan, Iran

**Keywords:** DNA Fragmentation, Fertilization, Globozoospermia, Male Infertility

## Abstract

**Objective:**

Phospholipase C zeta 1 (PLCζ) is one of the main sperm factor involved in oocyte activation and other factors may assist this factor to induce successful fertilization. Microinjection of recombinant tr-kit, a truncated form of c-kit receptor, into metaphase II-arrested mouse oocytes initiate egg activation. Considering the potential roles of tr- KIT during spermiogenesis and fertilization, we aimed to assess expression of tr-KIT in sperm of men with normal and abnormal parameters and also in infertile men with previous failed fertilization and globozoospermia.

**Materials and Methods:**

This experimental study was conducted from September 2015 to July 2016 on 30 normozoospermic and 20 abnormozoospermic samples for experiment one, and also was carried out on 10 globozoospermic men, 10 men with a history low or failed fertilization and 13 fertile men for experiment two. Semen parameters and sperm DNA fragmentation were assessed according to WHO protocol, and TUNEL assay. Sperm tr- KIT was evaluated by flow cytometry, immunostaining and western blot.

**Results:**

The results show that tr-KIT mainly was detected in post-acrosomal, equatorial and tail regions. Percentage of tr-KIT-positive spermatozoa in abnormozoospermic men was significantly lower than normozoospermic men. Also significant correlations were observed between sperm tr-KIT with sperm count (r=0.8, P<0.001), motility (r=0.31, P=0.03) and abnormal morphology (r=-0.6, P<0.001). Expression of tr-KIT protein was significantly lower in infertile men with low/ failed fertilization and globozoospermia compared to fertile men. The significant correlation was also observed between tr-KIT protein with fertilization rate (r=-0.46, P=0.04). In addition, significant correlations were observed between sperm DNA fragmentation with fertilization rate (r=-0.56, P=0.019) and tr-KIT protein (r=-0.38, P=0.04).

**Conclusion:**

tr-KIT may play a direct or indirect role in fertilization. Therefore, to increase our insight regarding the role of tr-KIT in fertilization further research is warranted.

## Introduction

Intra-cytoplasmic sperm injection (ICSI) technique has been applied increasingly to treat sperm-related infertility. During this technique, natural sperm selection barriers present in female reproductive tract and also initial physiological process of fertilization such as capacitation and acrosome reaction are bypassed. Therefore, it allows couples with little hope of achieving successful pregnancy to acquire fruitful fertilization and pregnancy (1- 4). Despite this potential, low or complete fertilization failure still occurs in a considerable number of ICSI cases. This phenomenon is mainly related to inability of sperm to induce oocyte activation (5, 6). In this regard, Swain and pool (6) showed that over 50% etiology of failed fertilization post in vitro fertilization (IVF) is related to failed oocyte activation and so far several sperm factors are described to be involved in oocyte activation, including: testis-specific phospholipase C zeta 1 (PLCζ), postacrosomal sheath WW domain-binding protein (WBP2NL or PAWP) and truncated c-kit gene product (*tr-KIT*) (7-12). These factors, commonly termed "spermborne oocyte activation factors (SOAFs)", are released into ooplasm upon fusion of sperm with oocyte and lead to intracellular calcium oscillation (13).

A large body of consistent and reproducible evidence suggests that PLCζ is the main factor that instigate Ca^2+^ release from intracellular stores (11, 13-17) and other factors may assist this protein in this process. Evidence may suggest that WBP2NL/PAWP may complement action of PLCζ by activating PLCγ noncanonically (7, 8, 18-21). Possibly, PAWP acquires this action via Yes- Associated Protein (YAP) which has an SH3 binding motif and this motif interacts with an SH3 domain in PLCγ. Therefore, Ca^2+^ release from intracellular stores via the PIP2 vesicles is initiated. Even though this signaling pathway has been envisaged for PAWP, but further verifications are required (10, 22). Similarly, shortened cytoplasmic product of *c-KIT*, called tr- KIT activate Fyn (a Src-like kinase) and subsequently SH3 binding motif of this kinase interacts with the SH3 domain in PLCγ to induce Ca^2+^ release from intracellular stores (22, 23). In this regard, Rossi et al. (22) suggested "microinjection of tr-kit into mouse eggs causes their parthenogenetic activation (12, 23). Thus, tr-KIT is a candidate as an assistant sperm factor that might play a role in the final function of the gametes, fertilization." However, further verification of these pathway remains to be explored.

## Materials and Methods

### Ethical approval

 This experimental study has been approved by the Ethics Committee of Royan Institute (94000127). Written consent was obtained from all patients and their partners included in this study.

### Experiment one: assessment of tr-KIT in individuals with normal and abnormal semen parameters

#### Study population and semen samples analysis

Ejaculated semen was obtained from 50 men who were referred for semen analysis to the Andrology Unit of Isfahan Fertility and Infertility Center (IFIC). Semen parameters were assessed according to WHO (27) protocol and semen samples were considered as normal or abnormal according to the WHO-2010 criteria. Individuals with sperm concentration ≥15 million per ml, total sperm count of > 35 million per ejaculate, percentage total motility higher than 40% and/or percentage abnormal morphology of lower than 96% were considered as "normozoospermic" or "normal parameters" group. Based on this categorization 30 individuals were selected for normozoospermia group and 20 individuals with at least two abnormal sperm parameters were included in "abnormozoospermia" or abnormal parameters" group. Semen samples with greater than one million WBC or other cell types were also excluded from our study. The remaining semen samples were used for assessment of tr- KIT by flow cytometry.

#### Verification of expression of *tr-KIT* and *c-KIT*

**Fig.1:**
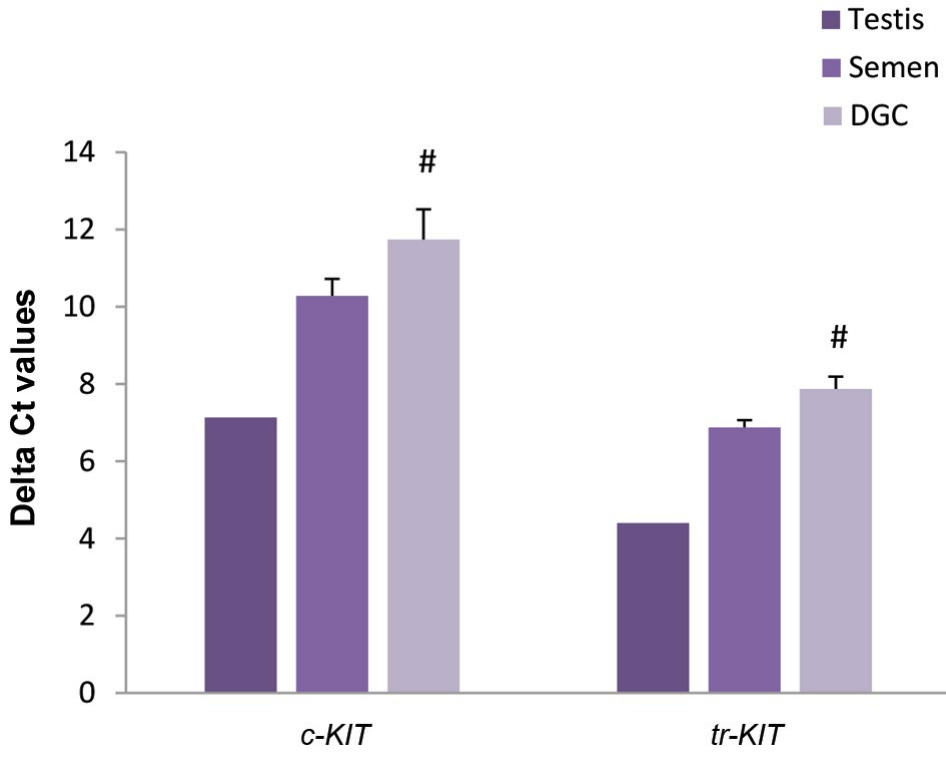
Assessment of c-KIT and tr-KIT transcripts in a testicular tissue, fresh semen (n=5), and DGC processed semen (n=5) samples. DGC; Density gradient centrifugation, and #; Shows a significant difference between fresh semen and DGC processed semen (n=5) samples at P<0.05.

In order to assess expression of *tr-KIT* and *c-KIT* in sperm, one pair of primer for *tr-KIT* (phosphotransferase domain) and one pair of primer for *c-KIT* (ligand-binding domain), were designed. Then, expression of *tr-KIT* and c-KIT were assessed in one testicular biopsy from obstructive azoospermia undergoing ICSI, washed, and processed semen samples from normozoospermic individuals (n=5) by real time polymerase chain reaction (PCR) (Fig .1). In addition, western blot analyses were carried on sperm from fertile and infertile men in experimental two for detection of tr-KIT and c-KIT bands in sperm by an anti-human primary antibody (Santa Cruz, USA).

#### Assessment of tr-KIT by flow cytometry

Briefly, semen samples from both groups were washed twice in phosphate-buffered saline (PBS) and fixed in cold acetone. Then, sperm pellets were washed twice with PBS for 5 minutes at 3000 rpm and incubated with bovine serum albumin (BSA, Sigma-Aldrich, USA) 5%+normal goat serum (NGS, Chemicon, Germany) 10% for 2 hours to block non-specific binding sites. Next, the affinitypurified anti-human primary antibody (Santa Cruz, USA) in PBS containing 1% bovine serum albumin (BSA) was applied overnight at 4˚C [this antibody detect both tr-KIT and c-KIT bands at 150 and 30 kDa, according to previous published paper by Muciaccia et al. (26), respectively. The result of this study and previous studies (22) show that unlike tr-KIT, expression of c-KIT protein is not observable in sperm. Therefore, we used C-19 antibody for assessment of tr-KIT by flow cytometry in individuals with normal and abnormal semen parameters]. Subsequently, samples were washed with PBS and incubated with goat anti-rabbit IgG secondary antibody complexed with FITC (Sigma, USA) for 1 hour at 37˚C. Ultimately, samples were washed with PBS and stained with propidium iodide (1 μg/ml, Sigma-Aldrich, USA). The percentage of tr-KIT-positive spermatozoa, propidium iodide positive sperm- population was assessed by a FACSCalibur flow cytometer (Becton Dickinson, San Jose, CA, USA) by means of an argon laser with an excitation wavelength of 488 nm and an emission wavelength of 530 nm. The analysis was carried out with subtraction of the fluorescence of control sample from test sample. For each assay, a minimum of 10,000 sperm were examined and the data were analyzed using BD CellQuest Pro software. Assessment of tr-KIT was performed according to modified protocols by Muciaccia et al. (26). Similar procedure was used for determine of tr-KIT localization by fluorescence microscope and, for each sample negative control were prepared without primary antibody.

### Experiment two: assessment of tr-KIT expression and DNA fragmentation in fertile and infertile men with globozoospermia and failed fertilization

#### Study population and semen samples analysis

Freshly ejaculated semen was obtained from 33 fertile and infertile men attending the Andrology Unit of IFIC. Infertile men were divided two groups; globozoospermia [(100% round-headed without acrosome) (n=10)] and individuals with a history failed or low fertilization (n=10). Individuals with total fertilization failure were considered as “failed fertilization” and those with fertilization rate of lower than 25% were considered as “low fertilization”. Thirteen fertile individuals who were referred for embryo donation or family balancing were considered as fertile or control group. For globozoospermia, protein was obtained from Royan protein bank (September 2013 to July 2016). For this study, individuals with failed and low fertilization were asked to voluntarily produce a second semen sample, within 7 days following ICSI. The remaining semen sample from individuals referred for embryo donation or family balancing was also used. Fertilization was assessed by the presence of pronuclei around 16-18 hour post-ICSI. The fertilization rate was calculated from the ratio of fertilized oocytes to the total number of survived injected metaphase II oocytes, multiplied by 100.

After liquefaction of semen at room temperature, each sample was divided into two parts. The first portion was used immediately for semen analysis and assessment of sperm DNA fragmentation using TUNEL assay. For fertile individuals a portion of semen sample was initially used for density gradient centrifugation (DGC) processing for ICSI/IVF and the remaining portion was used for semen analysis and TUNEL assay. All the studied samples had somatic cell count of less than one million per ml. The second portion was used for western blot after washing with PBS and centrifugation at 220 g for 10 minutes. The cell pellet was used for protein extraction.

#### Assessment of sperm DNA fragmentation

Assessment of DNA fragmentation in sperm sample were carried out by a detection kit (Apoptosis Detection System Fluorescein, Promega, Germany). Briefly, sperm concentration was assessed and samples were washed in PBS. Then, 20-40 μl of washed sperm was smeared onto slides and fixed in 4% paraformaldehyde for 30 minutes at room temperature. After washing the slides, the sperm were treated with 0.2% Triton X-100 (Merck, Germany) for 5 minutes and washed twice in PBS again. The samples were covered with cover slips. For each sample, 200 randomly spermatozoa were counted using an Olympus fluorescence microscope (BX51, Japan) with the appropriate filters (460-470 nm) at ×100 magnification. The percentage of green fluorescing sperm (TUNEL positive) as fragmented DNA in sperm was reported (28).

### RNA isolation and quantitative real-time polymerase chain reaction

For RNA extraction and cDNA synthesis, we used Aghajanpour et al. (29) protocol. Briefly, semen samples were washed with PBS, and total RNA was extracted using Trizol (Sigma-Aldrich, USA) for both fertile and infertile samples. For removing contamination of genomic DNA, samples were treated with DNaseI (Fermentas, USA). 1 μg of total RNA were used for cDNA synthesis by random hexamer primer and the RevertAid ™H Minus First Strand cDNA. For Realtime PCR, we used a Step One Plus thermal cycler [Applied Biosystems (ABi)], and this method was carried out according to the manufacturer’s protocol (TaKaRa, Ohtsu, Japan). The PCR mixture contained 3 pmol/μl of each primer, 10 μl SYBR premix Ex Taq II (TaKaRa, Ohtsu, Japan), and 25 ng cDNA adjusted to a final volume of 20 μl using dH_2_O for each reaction. All reactions were carried out in triplicate. Real-time specific primer pairs were designed by the Beacon Designer 7.5. The primers used were previously designed as:

tr-KITF 5ˊ-CAGCCAGAAATATCCTCCTTACT-3ˊ (Exon 17)
  R: 5ˊ-GCCATCCACTTCACAGGTAG-3ˊ (Exon 18)
GAPDHF 5ˊ-CCACTCCTCCACCTTTGACG-3ˊ
  R: 5ˊ-CCACCACCCTGTTGCTGTAG-3ˊ
c-KITF 5ˊ-GCGAGAGCTGGAACGTGGAC-3ˊ (Exon 1)
  R: 5ˊ-CTGGATGGATGGATGGTGGAGAC-3ˊ (Exon 2)

The real time PCR protocol was carried out according to Tavalaee NasrEsfahani (30). For presentation of data, the CT of target mRNA was normalized by CT of the reference gene (*GAPDH*). The data was expressed as ΔCT (CT of target gene-CT of *GAPDH* gene). A sample with lower ΔCT indicate higher concentration of target mRNA and vice versa.

### Western blot technique

Expression of tr-KIT at protein level was assessed by western blot technique in 13 fertile, 10 infertile men with failed fertilization and, 10 globozoospermic men. Briefly, semen samples were washed with PBS, and extraction of protein was carried using TRI Reagent (Sigma-Aldrich, USA). Then, protein concentration was evaluated by Bradford assay (Bio-Rad, USA). 40 μg of protein for each sample were subjected to 12% sodium dodecyl sulfatepolyacrylamide gel electrophoresis (PAGE), and then transferred to polyvinylidene fluoride [(PVDF membrane (BioRad, USA)]. For GAPDH and tr-KIT, membranes were blocked with 10% skim milk at 4˚C overnight and, 5% skim milk at 4˚C for 1 hour, respectively. Then, membranes were incubated with primary antibodies [GAPDH for 90 minutes, and tr-KIT for overnight at room temperature, and 4˚C, respectively]. Then, membranes were washed and incubated with secondary antibodies (goat anti-rabbit IgG-HRP and Peroxidase-Conjugated Goat Anti-Mouse Immunoglobulins) for 1 hour (30). Densitometric analysis of the bands was performed by Quantity One Software v 4.6.9 (Bio-Rad, Germany). The data was normalized as mean intensity of the infertile’s band/mean intensity of fertile bands, and results were expressed as mean relative intensity.

### Data analysis

In this study, we used Microsoft Excel and SPSS (Version 17, Chicago, IL, USA) for data analyses. Equality of variances, and normal distribution were analyzed using Levene’s test and Shapiro-Wilk, respectively. Comparison of study variations between two groups were determined with independent-samples t test while between three groups were analyzed with one-way analysis of variance (ANOVA). In addition, pearson analysis was used to assess the correlations between difference parameters. Data were expressed as mean ± standard error of mean (SEM). A P<0.05 was statistically significant.

## Results

### Verification of expression of *tr-KIT* and *c-KIT* transcripts

Real time PCR results revealed that both c-KIT and tr-KIT transcripts are expressed by testicular tissue and sperm (Fig .1). For detection of tr-KIT and c-KIT proteins in sperm, we used C-19 antibody, according to previous published paper by Muciaccia et al. (26). The result of this study (please see experiment two) and previous studies (22) show that unlike tr-KIT, expression of c-KIT protein is not observable in sperm. Therefore, we used C-19 antibody for assessment of tr-KIT by flow cytometry in individuals with normal and abnormal semen parameters.

### Experiment one: assessment of tr-KIT in men with normal parameters and abnormal parameters

Following semen analyses of couples referred to IFIC, samples were considered as abnormal (n=20) or normal (n=30) based on WHO criteria. Mean values for sperm concentration (24.15 ± 4.76 vs. 73.84 ± 5.48), total sperm count (100.58 ± 23.93 vs. 341.16 ± 31.67), and percentage sperm motility (36.47 ± 3.78 vs. 62.16 ± 2.19) were compared between the two groups and were significantly lower in men with abnormal semen parameters compared to men with normal semen parameters. In addition, percentage of abnormal sperm morphology (98.47 ± 0.20 vs. 95.53 ± 0.23) was significantly higher in men with abnormal semen parameters compared to men with normal semen parameters.

**Fig.2:**
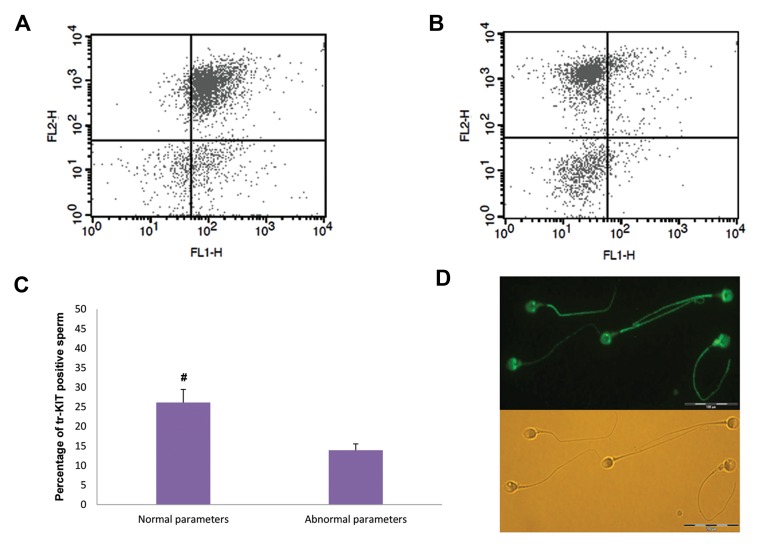
The results of flow cytometry and immunofluorescence staining of tr-KIT in sperm. **A.** Flow cytometric dot plot of tr-KIT in a man with normal, **B.** Abnormal semen parameters, **C.** Comparison of mean percentage of tr-KIT positive-spermatozoa between men with normal and abnormal semen parameters, and **D.** Localization of tr-KIT in sperm was evaluated by immunostaining method. **#;** Shows a significant difference between two groups at P<0.05.

**Fig.3:**
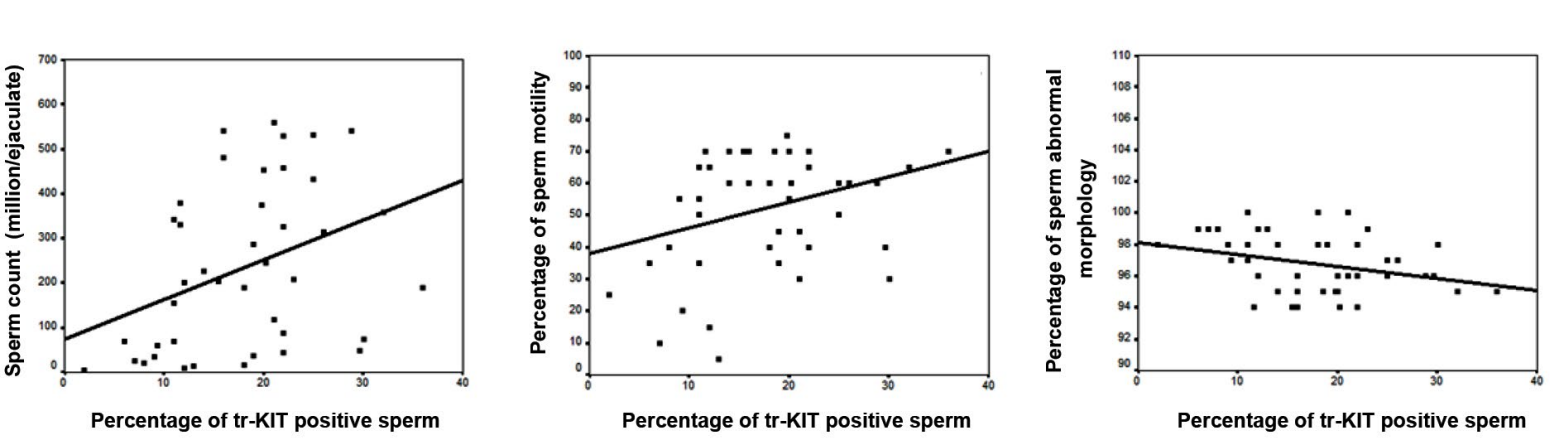
Correlation between percentage of tr-KIT positive-spermatozoa with sperm count (r=0.792, P<0.001), percentage of sperm motility (r=0.316, P=0.034), and sperm abnormal morphology (r=-0.595, P<0.001, n=50).

We assessed percentage of tr-KIT positive-spermatozoa by flow cytometry. Figure 2 shows that mean percentage of tr-KIT positive-spermatozoa was lower in men with abnormal semen parameters compared to men with normal semen parameters (Fig .2C, which quantifies Fig.2A, B). Localization of tr-KIT in sperm was evaluated by immunostaining method and we show that tr-KIT was mainly localized in the equatorial region of spermatozoa head and tail (Fig .2D), while tr-KIT was not detectable in these regions of spermatozoa in negative control. In addition, we observed significant correlations between percentage of tr-KIT positive-spermatozoa in the total population with sperm count (r=0.792, P<0.001), percentage sperm motility (r=0.316, P=0.034) and abnormal morphology (r=-0.595, P<0.001, Fig .3).

### Experiment two: assessment of sperm parameters, expression of sperm tr-KIT, and DNA fragmentation in fertile individuals, men with failed fertilization, and globozoospermia

#### Assessment of sperm parameters between fertile and infertile men

The mean values for female and male age were 34.4 ± 9.6 and 38.75 ± 5.37 in the fertile group; 26.4 ± 5.59 and 30.00 ± 3.93 in globozoospermia group and, 33.78 ± 2.16 and 38.45 ± 3.84 in couples with failed fertilization, respectively. In addition, sperm parameters were compared between groups. The mean values of sperm concentration were 73.76 ± 5.81, 37.89 ± 9.34 and 30.85 ± 13.62 in fertile, infertile men with failed fertilization, and globozoospermia groups, respectively. Means of sperm concentration were significantly reduced in men with failed fertilization and globozoospermia compared to fertile individuals (P<0.001). Furthermore, means percentages sperm motility were 61.53 ± 2.07, 36.67 ± 6.45, and 22.16 ± 9.29, in the fertile, infertile men with failed fertilization, and globozoospermia groups, respectively. Similar to sperm concentration, means of percentage of sperm motility were also significantly lower in the both infertile groups compared to the fertile group. In addition, percentage of sperm abnormal morphology was significantly higher in the infertile men with globozoospermia (100%) compared to fertile (95.3 ± 0.32) and infertile men with failed fertilization (96.34 ± 0.89) groups.

### Comparison of relative expression of sperm tr-KIT protein between fertile and infertile men

**Fig.4:**
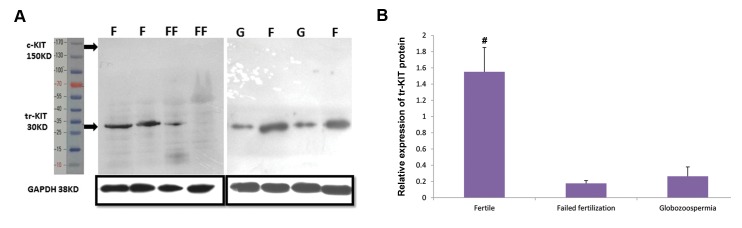
The results of western blots technique. **A.** Western blots of sperm tr-KIT protein from two fertile men. F; Fertile, FF; Two infertile men with failed fertilization, and G; Two infertile men with globozoospermia and **B.** Comparison of relative expression of tr-KIT protein between fertile men and both infertile groups. Arrows indicate bands of 150, 30 and 38 kDa for c-KIT, tr-KIT and GAPDH, respectively. **#;** Shows significant difference between fertile men and both infertile groups at P<0.05.

We also assessed relative expression of sperm tr-KIT protein by western blot technique in fertile, infertile men with failed fertilization and globozoospermic men. As shown in Figure 4, the band intensity of tr- KIT protein was low in infertile men with failed fertilization and globozoospermia compared to fertile individuals. Considering tr-KIT is a shortened protein produced by alternative splicing of c-kit, we used an antibody that able to detect both c-KIT and tr-KIT protein at 150 and 30 kDa bands. Our result was similar to Rossi et al. (22), and we did not observe any band at 150 kDa band in sperm, while tr-KIT was detectable. We compared mean relative expression of tr-KIT protein among these groups (Fig .4). The mean of tr-KIT protein was significantly lower in infertile men with failed fertilization (0.17 ± 0.03) and globozoospermic men (0.26 ± 0.12) compared to fertile (1.7 ± 0.5) men (P<0.05). In addition, we observed a significant correlation between fertilization rate with relative expression of tr-KIT protein (r=0.46, P=0.04).

### Comparison of sperm DNA fragmentation between fertile and infertile men

In this study, sperm DNA fragmentation was assessed by TUNEL assay in fertile and infertile men. Mean percentage of sperm DNA fragmentation was significantly higher in infertile men with failed fertilization (28.18 ± 6.01) and globozoospermic men (23.6 ± 5.67) compared to fertile (5.24 ± 1.23) groups (P<0.05). In addition, we observed negative significant correlations between percentage of DNA fragmentation with fertilization rate (r=-0.45, P=0.01), and tr-KIT protein (r=-0.38, P=0.04).

## Discussion

One of the cornerstones of development is ability of sperm to induce "oocyte activation". This event can initiate a series of physiological phenomena and metabolic reactions in oocyte such as release of calcium from intracellular stores, cortical granule exocytosis, block to polyspermy, and resumption of the meiotic cell cycle (15, 16). In addition to PLCζ as main factor involved in oocyte activation, several other sperm factors suggested that may assist PLCζ in this phenomenon (11, 9, 13). In this regard, several lines of evidence suggested low expression or absence of sperm factors involved in oocyte activation such as PLCζ and PAWP in men with low or failed fertilization post ICSI or globozoospermia (8, 29-35). Though, results of studies regarding the role of PAWP on fertilization and early embryonic development are still controversial. In this regard, Escoffier et al. (36) demonstrated that PLCζ alone is sufficient to induce oocyte activation. Among sperm factors, tr-KIT need to receive more attention in the field of male infertility.

In the mice model, previous studies showed that tr-KIT plays an important role in egg resumption from meiosis II at fertilization and zygotic development (25). In the light of these considerations, we decided to assess tr-KIT in human sperm. Our results clearly showed that both c-KIT and tr-KIT transcripts are present in testicular tissue and washed semen samples but western blot analysis revealed that only tr-KIT is present in sperm. Therefore, we assessed the percentage of tr-KIT positive sperm by flow cytometry in washed semen from individuals with normal and abnormal semen parameters. Our results showed that percentage of tr-KIT-positive spermatozoa was significantly lower in sperm of men with abnormal semen parameters compared to men with normal semen parameters. Also, significant correlations were observed between percent of tr-KIT-positive spermatozoa with sperm concentration, motility, and morphology. In this regard, Muciaccia et al. (26) also observed significant correlations between percentage of sperm tr-KIT with sperm motility and morphology, but not sperm count. The difference between current study with Muciaccia et al. (26), was due to sample sizes, type of selection of patient, and used technique. Considering expression of tr-KIT is restricted to spermiogenesis phase (25), therefore defects in spermatogenesis may be lead to misregulation of expression of this protein and subsequently cause poor semen quality. In line with this concept, other studies showed that misregulation of testis-specific genes could affect spermatogenesis and thereby semen quality (29, 37, 38). Therefore, assessment of sperm tr-KIT could be considered as an additional parameter along with classic semen analysis for evaluation of semen quality.

In addition, immunostaining results show that tr-KIT is localized in the post-acrosomal and equatorial regions. This observation is in of keeping with a previous study that stated "tr-kit is not present in soluble portion of sperm, but it is found mostly in the Triton-X100 insoluble material" by western blot analysis (39). Despite this claim, we also observed tr-KIT on sperm tail region. Considering that the percentage of tr-KIT-positive spermatozoa correlated significantly with sperm motility, we explain that presence of tr-KIT on sperm tail may be due to secondary role of this protein in physiological phenomenon like motility and capacitation and/or signaling pathway leading to oocyte activation. However, further research is needed to confirm these results.

In the next step, we assessed tr-KIT protein in sperm of infertile men with failed fertilization and globozoospermia. Relative expression of tr-KIT protein was significantly lower in infertile men with failed fertilization and globozoospermic individuals compared to fertile men. Therefore, low expression of tr-KIT could be along with many other proteins whose expression is reduced in infertile men with low or absence of fertilization. These data possibly could suggest that one reason of failed oocyte activation or failed fertilization in both infertile groups could be due to reduced expression of tr-KIT.

Considering that fertilization is a multifactorial process and many factors such as anomalies in the oocyte and/or sperm, chromatin damage, inability to activate the oocyte, failure in chromatin decondensation (6) and the technique used, could affect fertilization outcome, we assessed sperm DNA fragmentation in fertile individuals, and infertile men with either failed fertilization or globozoospermia. Sperm DNA damage that commonly due to oxidative stress has been associated with failure in fertilization, embryo quality as well as poor implantation, and pregnancy outcomes (40). As we expected, the percentage of fragmented DNA in the sperm of both infertile groups were higher compared to fertile men. In addition, we observed significant correlation between percentage of DNA fragmentation and fertilization rate. Therefore, individuals with high DNA fragmentation are likely to have low fertilization rates. Similar to Muciaccia et al. (26), we also observed a significant negative correlation between percentage of sperm DNA fragmentation and tr- KIT. Therefore, we suggest that sperm tr-KIT and DNA fragmentation could be considered as new markers for assessing human semen quality.

## Conclusion

The result of this study clearly demonstrated that sperm tr-KIT has an important association with fertilization in humans, and its expression is decreased in individuals with low fertilization rate and globozoospermia. Taken together, further studies are requiring to reveal more light on involvement of tr-KIT in fertilization and to deepen our undemanding regarding the molecular mechanism of failed fertilization.
